# 3 β-Hydroxysteroid-Δ 24 Reductase (DHCR24) Protects Neuronal Cells from Apoptotic Cell Death Induced by Endoplasmic Reticulum (ER) Stress

**DOI:** 10.1371/journal.pone.0086753

**Published:** 2014-01-29

**Authors:** Xiuli Lu, Yang Li, Weiqi Wang, Shuchao Chen, Ting Liu, Dan Jia, Xiaoping Quan, Deliang Sun, Alan K. Chang, Bing Gao

**Affiliations:** 1 The School of Life Science, Liaoning University, Shenyang, China; 2 School of Basic Medical Sciences, Shenyang Medical College, Shenyang, China; University of S. Florida College of Medicine, United States of America

## Abstract

3β-Hydroxysteroid-Δ24 reductase (DHCR24) is an endoplasmic reticulum (ER)-localized multifunctional enzyme that possesses anti-apoptotic and cholesterol-synthesizing activities. Accumulating evidence suggests that ER stress is involved in the pathogenesis of neurodegenerative disease. In this study, we investigated whether DHCR24 may function as a neuroprotective protein under ER stress. Neuroblastoma N2A cells were infected with adenovirus expressing myc-tagged DHCR24 (Ad-DHCR24) or lacZ (Ad-lacZ, serving as a control) and subjected to ER-stress, induced with Tunicamycin (TM). Cells infected with Ad-DHCR24-myc were resistant to TM-induced apoptosis, and showed weaker level of caspase-12 activity. These cells also exhibited lower levels of Bip and CHOP proteins than Ad-LacZ-infected cells. Moreover, a stronger and rapid activation of PERK, and a prolonged activation of JNK and p38 were observed in Ad-LacZ–infected cells. The generation of intracellular reactive oxygen species from ER stress was also diminished by the overexpression of DHCR24. Additionally, intracellular cholesterol level was also elevated in the Ad-DHCR24-infected cells, accompanied by a well-organized formation of caveolae (cholesterol-rich microdomain) on the plasma membrane, and improved colocalization of caveolin-1 and insulin-like growth factor 1 receptor. These results demonstrated for the first time that DHCR24 could protect neuronal cells from apoptosis induced by ER stress.

## Introduction

The endoplasmic reticulum (ER) is the site where proteins destined for the cell surface and endomembrane system enter the secretory pathway. Newly synthesized secretory and membrane-associated proteins undergo disulfide-bond formation and isomerization in the ER to yield correctly folded and assembled proteins. Under physiological condition, ER-protein load and protein-folding capacity achieves an equilibrium state. Changes in ER homeostasis due to increased protein synthesis, accumulation of misfolded proteins, or alterations in the calcium or redox balance of ER lead to a condition called ER stress [1], [2]. To cope with this stress, the cells have developed an adaptive signaling pathway called the unfolded protein response (UPR) or ER stress response. If homeostasis is not restored, the UPR is chronically activated and leads to cell death (apoptosis) [3], [4].

Accumulating evidence indicates that pathological conditions that interfere with ER homeostasis will give rise to chronic activation of UPR, which contributes to the pathogenesis of many diseases, including neurodegenerative disorders, type 2 diabetes, atherosclerosis, liver disease, and cancer [5]–[7]. A more specific example of one such disorder is Alzheimer’s disease (AD). AD is a progressive neurological disorder characterized by a decline in cognitive processes, eventually leading to dementia [6], [8], [9]. The hallmarks of this disease include the accumulation of extracellular amyloid-β (Aβ) peptides and intracellular aggregates of phosphorylated tau proteins, along with the perturbation of calcium homeostasis and neuronal death [10]. Recent reports have indicated that UPR is activated in the brain of patients with AD. There is also increased expression of the ER chaperone Grp78 (which is indicative of UPR activation) in the brains of AD patients [11]. Additionally, autopsy studies have revealed increased phosphorylated (activated) PERK, eIF2α, and IRE1 in the brains of patients with AD, compared to specimens from subjects without the disease. UPR-positive staining is localized to the neurons, and not glial cells, which is consistent with a role for ER stress in AD pathogenesis [12].

DHCR24 (also known as hDiminuto/Seladin-1) is an enzyme that acts as a 3β-hydroxysteroid-Δ24 reductase, and its level has been found to decrease in the brain of AD patients. DHCR24 catalyzes the final step of cholesterol biosynthesis, which is the conversion of desmosterol to cholesterol [13]. In addition to its cholesterol-synthesizing activity, several biologically important activities of DHCR24 have also been reported. Overexpression of DHCR24 protects neuronal cells from apoptosis induced by hydrogen peroxide or Aβ [14]. Furthermore, DHCR24 also interacts with and induces the accumulation of p53 [15]. It is thought that DHCR24 acts as an anti-apoptotic protein because reduced expression of the DHCR24 gene is associated with increased apoptosis of adrenocortical cells. We have previously cloned the DHCR24 gene, which is abundantly expressed in cortisol-producing adrenocortical adenomas [16]. Using mouse embryonic fibroblast cells (MEFs) obtained from *DHCR24* ^−/−^ mice, we demonstrated that DHCR24 can directly scavenge H_2_O_2_, protecting these cells from oxidative-stress-induced apoptosis [17]. Therefore, DHCR24 is a multifunctional protein, possessing both cholesterol-synthesizing and anti-apoptotic activities. However, the molecular mechanisms underlying its anti-apoptotic activity are not fully understood.

Utilizing the database obtained from Allen institute for brain science, we found that DHCR24 is mainly expressed in the anterior orbital gyrus and hippocampus area, suggesting that it has an important role in neuron function associated with learning and memory. A recombinant adenovirus driving the overexpression of DHCR24 (Ad-DHCR24) used in a previous study has shown that overexpression of DHCR24 protects MEFs from apoptosis initiated by the ER stress inducer, Tunicamycin [17]. To elucidate the roles of DHCR24 in neuronal cells, the current study investigated the neuroprotective function of DHCR24 under ER stress, and found that DHCR24 protected neuronal cells from ER stress-induced apoptosis by attenuating ER stress signaling, possibly through scavenging intracellular reactive oxygen species and elevating cholesterol levels.

## Materials and Methods

### Cell Culture

N2A cells (American Type Culture Collection, Manassas, VA) were cultured in DMEM/F12 (high glucose) supplemented with 10% fetal bovine serum. In some experiments, cells were treated with 1 µM Akt inhibitor IV (Calbiochem, San Diego, CA). Cell images were obtained with a phase-contrast microscope (IMT-2, Olympus, Tokyo, Japan) equipped with a digital camera (PDMC II, Polaroid, Waltham, MA).

### Analysis of Adherent Cell Numbers and Apoptosis Assay

Detached cells were removed from the culture plate by washing the plate once with medium. The adherent cells were trypsinized and collected by centrifugation. They were then stained with Trypan Blue using Trypan Blue-Staining Cell Viability Assay Kit (Biyuntian, Shanghai, China). The numbers of cells from different treatments were counted and compared.

DNA fragmentation was analyzed by the terminal deoxynucleotidyl transferase-mediated deoxyuridine triphosphate (dUTP)-biotin nick end labeling (TUNEL) method (Takara, Otsu, Japan) according to the manufacturer’s protocol.

Cellular apoptosis was assessed by *in situ* DNA fragmentation and immunocytochemistry-based caspase-3 assay. The procedure for immunocytochemical analysis was described previously [17], [18]. Briefly, after fixation and blocking, the cells were incubated with rabbit antibody directed against active caspase-3 (Sigma-Aldrich, St. Louis, Missouri, USA) followed by incubation with anti-rabbit IgG antibody conjugated to Alexa Fluor 568 (Molecular Probes, Eugene, OR). For the detection of Alexa fluor-568 fluorescence, the main beam splitter for excitation, the secondary beam splitter for emission, and barrier filter were set to 568 nm, 570 nm, and 585 nm long pass, respectively. Several images were captured with the same set of optical parameters. Densitometric analysis was performed using Multi Gauge software in LAS-1000 (Fuji Film).

### Determination of Intracellular Cholesterol

Lipid was extracted by the method of Bligh and Dyer [19]. Total sterol content in the lipid was determined by measuring the content of 3β-hydroxysterols using an enzymatic cholesterol assay kit (Roche Diagnostics, Mannheim, Germany).

### Western Blot Analysis

Whole cell lysate for Western blot analysis was prepared as previously described [17], [18]. For Western blot analysis, whole cell lysate (50 µg protein/lane) was first subjected to SDS-PAGE using 10% gel. After that, the proteins in the gel were transferred onto polyvinylidene difluoride membrane (Amersham Pharmacia, Piscataway, NJ). The blot was probed with the appropriate primary antibody as described below, followed by incubation with horseradish peroxidase-conjugated anti-rabbit or anti-mouse IgG antibodies. Primary antibodies: Rabbit anti-phospho-p38 MAPK (T180/Y182), anti-caspase12, anti-cleaved caspase-3, anti-p-PERK, anti-p-Akt, anti-Akt and anti-phospho-JNK (T183/Y185) antibodies were purchased from Cell Signaling (Beverly, MA). Mouse monoclonal anti-Bip/GRP78 antibody was from BD Biosciences (Bedford, MA). Rabbit anti-CHOP (GADD153) and mouse anti-myc antibodies were from Santa Cruz Biotechnology (CA, USA). Rabbit anti-actin, anti-caspase-12, and anti-cleaved caspase-3 antibodies were from Sigma-Aldrich. Positive protein bands in the blot were visualized using enhanced chemiluminescence reagents (Pierce, Rockford, IL). The images of the blotted membranes were obtained by an LAS-1000 lumino-image analyzer (Fuji Film, Tokyo, Japan). Densitometric analysis was performed with the same instrument.

### Immunocytochemical Analysis

Confocal laser scanning microscopy was performed as previously described [20]. Briefly, after the fixation and blocking steps, the cells were incubated with a mixture of rabbit anti-caveolin-1 polyclonal antibodies (Santa Cruz Biotechnology, Santa Cruz, CA) and mouse anti-IGF-I β monoclonal antibody (BD Biosciences, Bedford, MA) for 60 minutes, and then with a mixture of anti-mouse IgG antibody conjugated to Alexa fluor-488 (Molecular Probes, Eugene, OR) and anti-rabbit IgG antibody conjugated to Alexa fluor-568 (Molecular Probes). In other experiments, the cells were incubated with antibody against cleaved caspase-3, full-length caspase-12, or CHOP followed by secondary antibody. Images were obtained using a confocal laser microscope (LSM510, Carl Zeiss, Jena, Germany). Several images were captured with the same set of optical parameters, and merged using Adobe photoshop software (ver. 7.0.1, Adobe Systems, San Jose, CA). All images were taken with a 40/1.4 numerical aperture (NA) oil differential interference contrast Plan-Neofluar (Zeiss) using a 488-nm Ar/Kr laser line or a 543- HeNe laser line. Scanning speed and laser intensity were adjusted to avoid photobleaching of the fluorophores and damage to the cells. The microscope was equipped with a microenvironmental chamber to maintain a physiological condition. For quantification of fluorescence intensity, nonsaturated images were taken with a full-open pinhole. For multichannel imaging, each fluorescent dye was imaged sequentially in the frame-interlace mode to eliminate cross-talk between the channels. Three independent experiments were performed for Ad-lacZ- and Ad-DHCR24-infected cells, and 40 cells were randomly captured from a single group with the same set of optical parameters and their fluorescence intensity was measured and calculated to obtain an average value per cell.

All image processing was performed using the Zeiss LSM 510 image examiner software.

### Measurement of ROS Production

Intracellular ROS production was measured by a fluorescent-dye technique [20]. N2A cells were cultured on glass cover-slips and then treated for 30 min with 20 µM 2′, 7′-dichlorofluorescin diacetate (DCFHDA, Molecular Probes) in phosphate buffered saline (PBS). The cover slips were fixed and mounted on a glass slide. To detect the fluorescence of 2′, 7′-dichlorofluorescein, the main beam splitter for excitation, the secondary beam splitter for emission, and barrier filter were set at 488 nm, 570 nm, and 505 nm long pass, respectively. Several images were captured with the same set of optical parameters. Densitometric analysis was performed using Multi Gauge software in LAS-1000 (Fuji Film). Three independent experiments were performed for Ad-lacZ and Ad-DHCR24 infected cells, and 40 cells were randomly captured from a single group with the same set of optical parameters and their fluorescence intensity was measured and calculated to average value per cell.

### Statistical Analysis

Statistical analysis was performed with ANOVA followed by Bonferroni’s multiple t-tests, and statistical significances were considered at the *P*<0.05 level.

## Results

### Adenovirus-delivered DHCR24 Protects N2A Cells from Apoptosis Induced by ER Stress

**Figure 1:**
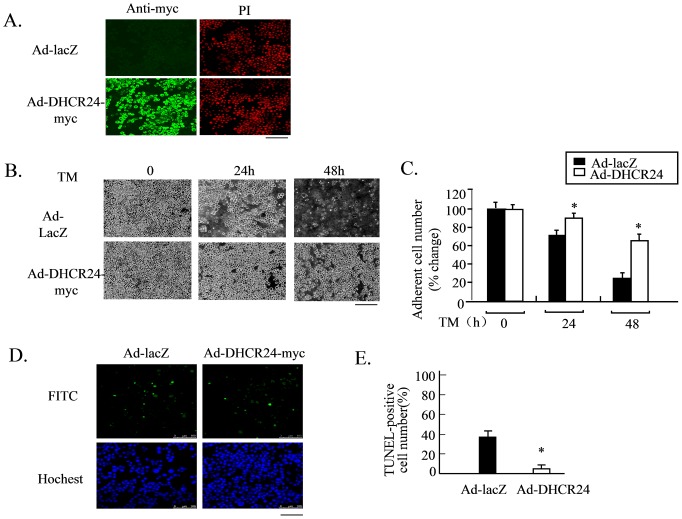
Overexpression of DHCR24 protects neuronal cells from TM-induced apoptosis. Panel A, The overexpression of DHCR24 in N2A cells was validated by immunocytochemical analysis using an antibody against c-myc. Cells were infected with adenoviruses for 48 hours and probed with mouse anti-myc antibody, followed by anti-mouse IgG antibody conjugated to Alexa fluor-488. Images were obtained by confocal laser microscopy. Green fluorescence signal represents the expression of recombinant DHCR24-myc and red fluorescence signal represents propidium iodide (PI)-stained nuclei. Scale bar, 100 µm. Panel B, N2A cells were infected with the indicated adenoviral construct at an MOI of 50 PFU/cell for 48 hours followed by exposure to TM (1 µg/ml). Images of the cells were obtained with a phase-contrast microscope at 0, 24 and 48 hours after TM treatment. Scale bar, 100 µm. Panel C, Numbers of adherent cells from panel B at 0, 24 and 48 hours. Values are expressed as mean ± S.D. (n = 3) *p<0.05 versus Ad-LacZ. Similar results were obtained from three separate experiments. Panel D, N2A cells were infected with the indicated adenoviral construct for 48 hours and then exposed to TM for 48 hours. DNA fragmentation was analyzed by the TUNEL assay and nuclei were stained with Hoechst. Scale bar, 100 µm. Panel E, The percentages of apoptotic cell numbers to adherent cells of Panel D were determined 48 hours after TM treatment. Values are expressed as mean ± S.D. (n = 3) *p<0.05 versus Ad-LacZ. Similar results were obtained from three separate experiments.

We have previously constructed a recombinant adenovirus to drive the expression of DHCR24 tagged with a myc epitope at the C-terminus (Ad-DHCR24), and demonstrated that DHCR24 expressed from Ad-DHCR24 can protect mouse embryonic fibroblasts from oxidative stress-induced apoptosis [17]. We first confirmed the transduction efficiency of Ad-DHCR24 in N2A cells by immunocytochemical analysis using an antibody against the myc tag. More than 85% transduction efficiency was achieved as revealed by the presence of DHCR24-myc in the cytoplasm of the infected cells (Fig. 1A and B). Adenovirus-driven expression of β-galactosidase (Ad-LacZ) was used as a negative control.

To induce ER stress, we used tunicamycin (TM), an inhibitor of N-linked glycosylation, which is widely used as an ER stress inducer. We found that N2A cells overexpressing Ad-DHCR24 were resistant to TM. After 48 hours of TM exposure, these cells still survived, with only insignificant loss of cells (Fig. 1B and C). In contrast, Ad-LacZ-infected cells were progressively lost. TUNEL assay showed that 24 hours after treatment with TM, roughly 40% of N2A cells infected with Ad-LacZ underwent apoptosis in response to TM, whereas virtually all N2A cells infected with Ad-DHCR24 were resistant to apoptosis (Fig. 1 D and E). These results demonstrated that overexpression of DHCR24 induced by adenovirus could protect neural cells from ER-stress-induced apoptosis.

### Overexpression of DHCR24 Inhibits Apoptotic Cell Signaling

**Figure 2:**
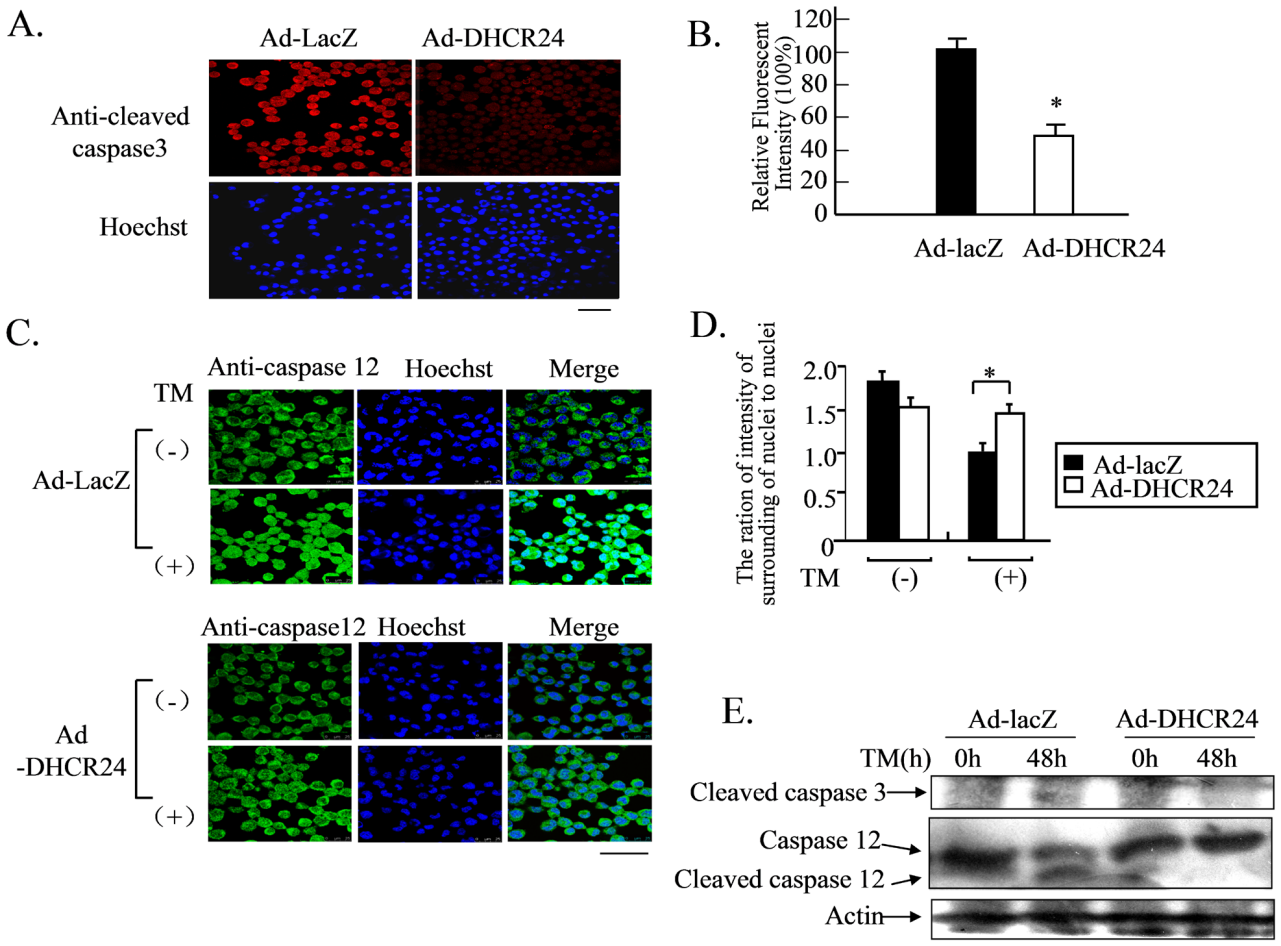
DHCR24 suppresses caspase-mediated apoptotic signaling pathway. Panel A, N2A cells were infected with the indicated adenoviral construct for 48 hours and then exposed to TM for 48 hours, followed by immunocytochemical analysis. The cells were probed with mouse antibodies against the cleaved form of caspase-3, followed by anti-mouse IgG antibody conjugated to Alexa fluor-568. The nuclei were stained with Hoechst. Images were obtained with a confocal laser microscope. Scale bar, 50 µm. Panel B, The relative fluorescence signal intensities of Panel A were represented as percentage of Ad-LacZ. Values are expressed as mean ± S.D. (n = 3) *p<0.05 versus Ad-LacZ. Similar results were obtained from three separate experiments. Panel C, N2A cells were infected with the indicated adenoviral construct for 48 hours and then exposed to TM for 0 [TM(−)] or 48 hours [TM(+)], followed by immunocytochemical analysis. The cells were probed with mouse anti-caspase-12 antibody, followed by an anti-mouse IgG antibody conjugated to Alexa fluor-488. Images were obtained with a confocal laser microscopy. Scale bar, 100 µm. Panel D, The ratios of fluorescence intensity outside the nuclei to that inside the nuclei in Panel C images are represented. Values are expressed as mean ± S.D. (n = 3) *p<0.05 vs. Ad-LacZ. Similar results were obtained from three separate experiments. Panel E, N2A cells were infected with the indicated adenoviruses for 48 hours and then exposed to TM for 0 or 48 hours. Whole cell lysates (50 µg protein/lane) were subjected to Western blot analysis using anti-cleaved caspase-3, anti-caspase-12 (both full-length and cleaved form), and anti-actin antibodies.

To confirm and identify the relevant apoptotic signaling pathway affected by the overexpression of DHCR24, both Ad-DHCR24-infected and Ad-LacZ-infected N2A cells cultured on cover slips were subjected to immunocytochemical analysis using an antibody against the activated (cleaved) form of caspase-3. The detection of active caspase-3 is widely used to detect the presence of apoptosis in many cell lines. As shown in Fig. 2 A and B, the number of Ad-LacZ-infected cells displaying intense fluorescence signals was significantly higher compared to Ad-DHCR24-infected cells. This result suggested that overexpression of DHCR24 inhibited the activation of caspase-3, thus blocking the progression and onset of apoptosis.

It has been reported that caspase-12 is an ER stress-specific caspase that mediates ER stress-induced apoptosis, and is activated during the disruption of ER calcium homeostasis. Caspase-12 is co-localized to the ER and mediates cytotoxicity induced by Aβ [21], [22]. Thus, we investigated whether caspase-12 was activated under TM-induced ER stress. We found that the enzyme was mainly localized to the ER of both Ad-LacZ- and Ad-DHCR24-infected cells when these cells were cultured in normal growth medium (Fig. 2 C TM (−)) (under non-ER-stress conditions), consistent with previous reports. However, when these cells were exposed to TM, the fluorescence signal of caspase-12 was more diffuse in Ad-LacZ-infected cells (indicative of translocation from the ER to the cytoplasm) than in Ad-DHCR24-infected cells, where translocation was inhibited. This suggested that overexpression of DHCR24 probably inhibited the caspase-12-mediated apoptotic signaling pathway that was strongly activated by TM in the control cells (Fig. 2 C TM (+)).

The difference in apoptotic pathway signaling exhibited by cells that did overexpress DHCR24 and cells that did not was confirmed by western blot using antibodies against both cleaved caspase-3 and full-length caspase-12. As shown in Fig. 2 E, cleaved caspase-3 was more abundant in Ad-LacZ infected cells than in Ad-DHCR24-infected cells 48 hours after TM treatment. Similar results were observed for the activation of caspase-12. Taken together, these results demonstrated that TM-induced ER stress led to the activation of caspase-mediated apoptosis, but this process could be blocked by overexpression of DHCR24.

### Overexpression of DHCR24 Reduces/delays Activation of ER Stress-related Cell Signaling during UPR in N2A Cells

**Figure 3:**
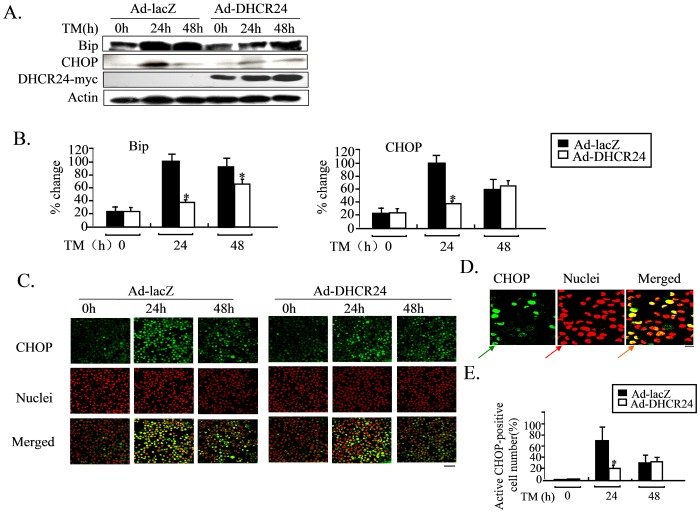
ER stress is less severe in Ad-DHCR24-infected N2A cells. N2A cells were infected with the indicated adenoviruses for 2 days, and then exposed to TM for various lengths of time. Whole cell lysates (50 µg/lane) were subjected to western blot analysis using anti-Bip, anti-CHOP, anti-myc or anti-actin antibody. The proteins were visualized using the ECL method. Representative results are shown in panel A. The expression levels of Bip and CHOP were normalized against actin level, and are expressed as percentage of the maximal level in the cells infected with Ad-LacZ. n = 3, Mean ± S.D., *: p<0.05 versus the levels in cells infected with Ad-LacZ. Cells were cultured on cover slips and infected with adenoviruses for 2 days, and then exposed to TM for the indicated time points, followed by immunocytochemical analysis. Cells were probed with mouse anti-CHOP antibodies, followed by anti-mouse IgG antibody conjugated to Alexa fluor-488. Nuclei were stained with PI. Images were obtained by confocal laser microscopy. Scale bar, 50 µm (Panel C). Scale bar, 5 µm (Panel D). The relative fluorescence signal intensities of Panel C were represented as percentage relative to that at time 0. Values are expressed as mean ± S.D. (n = 3) *p<0.05 versus Ad-LacZ. Similar results were obtained from three separate experiments.

It has been reported that Bip (immunoglobulin heavy-chain binding protein, Grp78) and CHOP (C/EBP homologous protein, CHOP) are upregulated and activated in response to ER stress [11], [23]. We next investigated whether there was any difference in the expression levels of Bip and CHOP between Ad-DHCR24-infected cells and control cells during TM-induced ER stress. Western blot analysis indicated that the levels of Bip and CHOP were upregulated in both Ad-DHCR24-infected and control cells after exposure to TM for 24 and 48 hours (Figs. 3A and 3B), confirming the presence of TM-induced ER stress. However, the upregulation of both Bip and CHOP was much weaker in Ad-DHCR24-infected cells. Even 48 hours after TM treatment, the level of DHCR24 in the cells was still strong, probably sufficient to protect the cells from TM-induced apoptosis. Immunocytochemical analysis showed that CHOP was almost un-detectable in both Ad-DHCR24- and Ad-LacZ-infected cells before TM treatments (Fig. 3C and D). Strong fluorescence signals were observed in the nuclei of most Ad-LacZ-infected cells in response to TM. In the case of Ad-DHCR24-infected cells, fewer cell nuclei exhibited similarly strong fluorescence signals after TM treatment. This suggested that overexpression of DHCR24 could suppress TM-induced translocation and activation of CHOP (Fig. 3 C, D and E).

**Figure 4:**
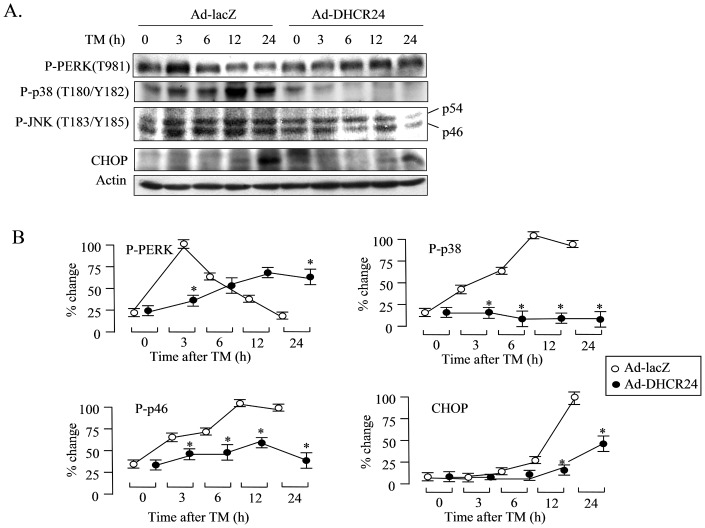
ER-stress-related cell signaling is suppressed in Ad-DHCR24-infected N2A cells. N2A cells were infected with the indicated adenoviruses for 2 days, and then exposed to TM for various lengths of time. Whole cell lysates (50 µg protein/lane) were subjected to western blot analysis using anti-phospho-PERK (T981), anti-phospho-p38 (T180/Y182), anti-phospho-JNK (T183/Y185), anti-CHOP or anti-actin antibody. Positive bands were visualized using the ECL method. Representative results are shown in panel A. The phosphor-PERK, phosphor-p38 and phosphor-JNK levels were normalized to actin levels, and are expressed as percentage relative to the maximal level in the cells infected with Ad-LacZ. n = 3, Mean ± S.D., *: p<0.05 vs. the levels in cells infected with Ad-LacZ.

Prolonged activation of stress-related signaling can induce apoptosis [3], [24], [25]. To further confirm the effect of DHCR24 overexpression on ER stress-related cell signaling, we investigated the activation of PERK, p-38 and JNK by western blot analysis. In the case of Ad-LacZ-infected cells, PERK was strongly activated as early as 3 hours after TM treatment (Fig. 4). However, the activation of PERK was delayed to 6 hours and was somewhat weaker in the case of Ad-DHCR24-infected cells. Similarly, the activation of p38 and JNK was significantly reduced in Ad-DHCR24-infected cells compared to the controls. Taken together, these results demonstrated that adenovirus-driven overexpression of DHCR24 protected neuronal cells from ER stress-induced apoptosis by delaying and reducing the activation of ER stress-related signaling at the early stage of ER stress.

### DHCR24 Scavenges Intracellular ROS during ER Stress

**Figure 5:**
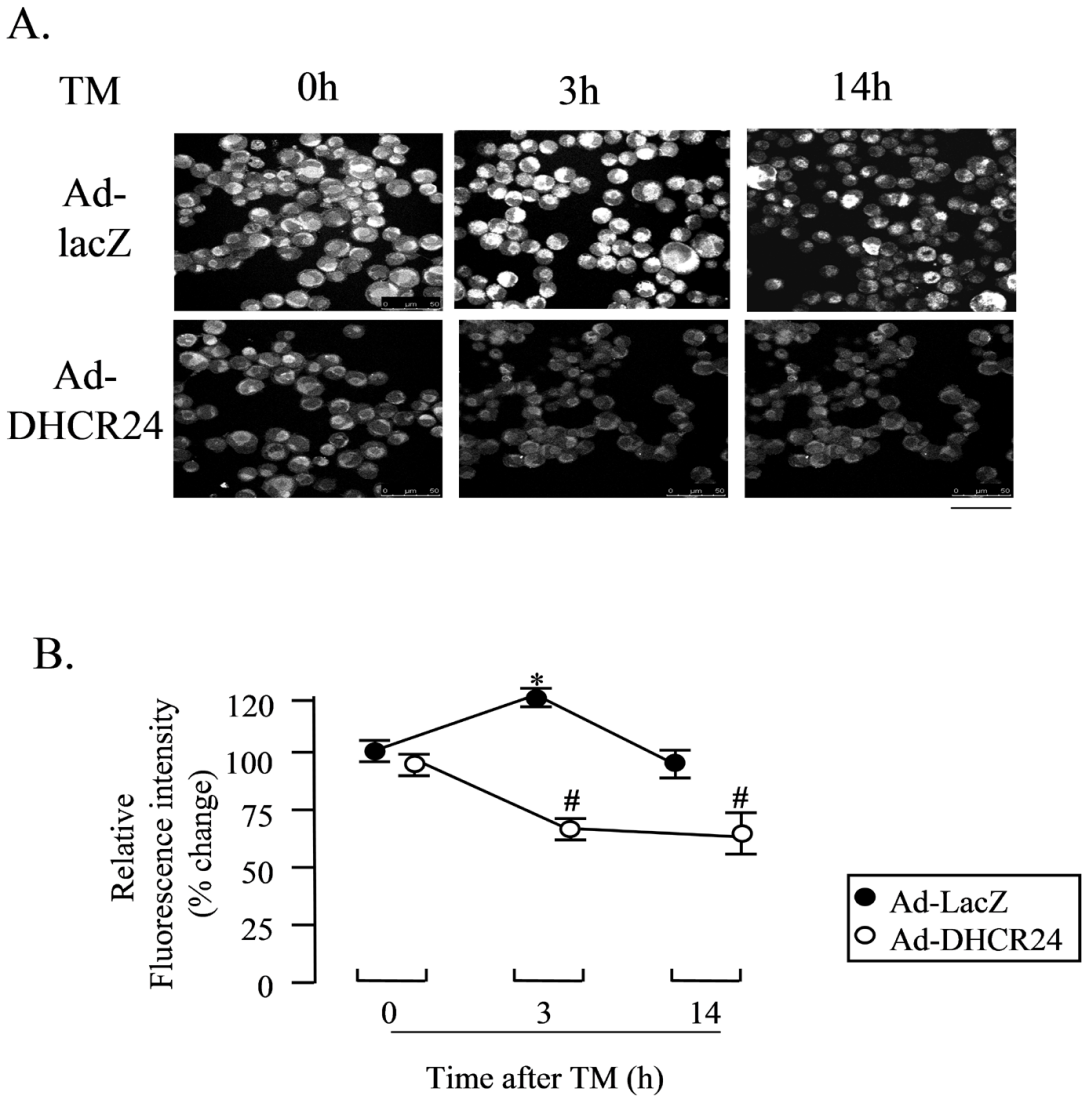
Overexpressed DHCR24 in N2A cells scavenges intracellular ROS under ER stress. N2A cells were infected with the indicated adenoviral construct for 2 days, and then exposed to TM for various lengths of time. The cells were subjected were treated with 20 µM H_2_DCFDA at 37°C for 30 minutes, and then fixed and mounted. Cell images were obtained with a confocal laser microscope. Bar, 50 µm (Panel A). Fluorescent intensity of DCF (2′, 7′ –dichlorofluorescein) was expressed relative to that at time 0. Mean ± SD (n = 40 cells). *: p<0.05 versus the levels of time 0 in cells infected with Ad-LacZ. #: p<0.05 versus the levels in cells infected with Ad-LacZ (Panel B).

Several studies have demonstrated that oxidative stress and the onset of ER stress are connected and that some antioxidants are able to reduce ER stress [3], [26], [27]. It has also been reported that loss of PERK can cause defects in the sensitivity to cell death under pathological conditions linked to ROS-mediated ER stress [3], [28]. We have previously reported that DHCR24 can protect mouse embryonic fibroblast from H_2_O_2_-induced apoptosis by scavenging intracellular ROS [17]. Thus, we investigated whether DHCR24 could scavenge ROS precipitated by ER stress in neuronal cells. Intracellular ROS was detected by DCFHDA, which was used as a fluorescent probe. As shown in Fig. 5, the fluorescence signal was stronger when the cells were exposed to TM for 3 hours compared to zero time treatment in the case of Ad-LacZ-infected cells, suggesting that the excess intracellular ROS was generated by ER stress. However, compared to Ad-LacZ-infected cells, Ad-DHCR24-infected cells showed a significantly weaker signal when the cells were exposed to TM for 3 hours, indicating the presence of a weaker oxidative environment. These data suggested that overexpression of DHCR24 suppressed the excess generation of intracellular ROS induced by ER stress, and this is consistent with our previous study [17].

### Elevated Cholesterol Levels Induced by Ad-DHCR24 May Contribute to the Neuroprotective Function of DHCR24

**Figure 6:**
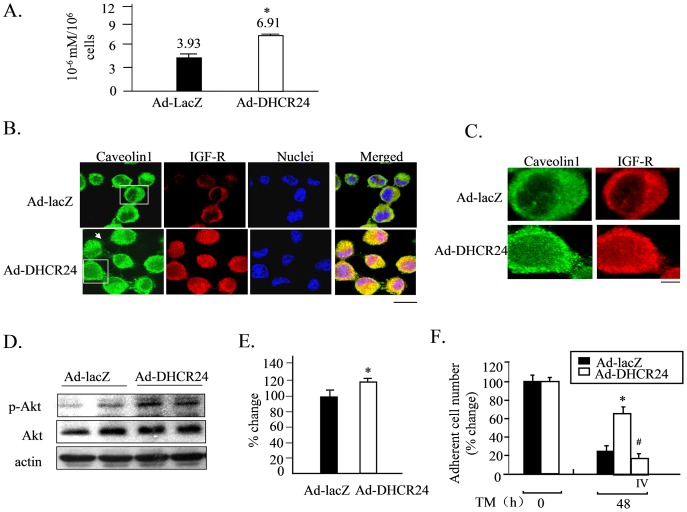
Elevated cholesterol levels induced by Ad-DHCR24 infection facilitates caveolae structural formation and coupling of IGFI-R and caveolin-1. Panel A, N2A cells were infected with the indicated adenoviruses for 2 days and the sterol contents in the whole cell extract were then determined using an enzymatic cholesterol assay kit. n = 3, Mean ± S.D., *: p<0.05 versus the levels in cells infected with Ad-LacZ. Panel B and C, N2A cells were infected with the indicated adenoviruses for 2 days, and then subjected to immunocytochemical analysis. The cells were probed with rabbit anti-caveolin-1 and mouse anti-IGF-IR β subunit antibodies, followed by anti-mouse IgG antibody conjugated to Alexa fluor-488 and anti-rabbit IgG antibody conjugated to Alexa fluor-568. Images were obtained with a confocal laser microscope. Red and green fluorescent signals represent caveolin-1 and IGF-IR proteins, respectively. Scale bar, 10 µm (Panel B), 1 µm (Panel C). Panel D and E, N2A cells were infected with Ad-lacZ or Ad-DHCR24 for 2 days, harvested, and then subjected to Western blot analysis using the anti-p-Akt, anti-Akt and anti-actin. Phosphor-Akt levels were normalized to actin level, and expressed as percentage of the levels in the cells infected with Ad-LacZ. n = 3, Mean ± S.D., *: p<0.05 versus the levels in cells infected with Ad-LacZ. Panel F, N2A cells were infected with the indicated adenoviruses for 2 days, and then exposed to the TM without or with Akt inhibitor IV (IV) for 48 hours. After trypsinization, the viability of the cells was assessed by Trypan Blue Staining. The number of viable cells is presented as a percentage of the number of cells infected with Ad-LacZ without TM treatment (n = 3, Mean±SD). *: p<0.05 versus cells infected with Ad-lacZ and treated with TM, #: p<0.05 versus cells infected with Ad-DHCR24 treated with TM.

As DHCR24 is involved in the conversion of desmosterol to cholesterol, we next measured the intracellular cholesterol levels of both Ad-LacZ and Ad-DHCR24-infected cells. The cholesterol level of Ad-DHCR24-infected cells was significantly elevated compared to that of Ad-LacZ-infected cells (Fig. 6A). Cholesterol in neuronal cell membranes plays an important role in maintaining the structure and function of caveolae/lipid rafts, the sites where many neuronal growth factors receptors (e.g., insulin-like growth factor (IGF-1) and neuron growth factor (NGF)) are located [29], [30]. We previously reported that cholesterol loss induced by double deletion of the *DHCRC24* gene can destroy the cellular localization of insulin receptor and impair the Akt-Bad cell survival signaling pathway in mouse embryonic fibroblasts [18], [31]. Thus the effect of DHCR24 overexpression on the structure of caveolae and location of IGF-1 receptor (IGF-1R) in N2A cells was examined by immunocytochemical analysis. As shown in Fig. 6B and C, the merging of green fluorescence (which represents caveolin 1(the molecular marker of caveolae)) and red fluorescence (which represents IGF-1R) yielded yellow fluorescence in Ad-LacZ-infected cells, confirming the localization of IGF-1R in the caveolae. The spots-like green and red signals were much stronger and more concentrated in Ad-DHCR24-infected cells compared to Ad-LacZ-infected cells. Western blot analysis was performed to evaluate the activation of Akt induced by overexpression of DHCR24. As shown in Figs. 6D and 6E, the phosphorylation of Akt was slightly stronger in Ad-DHCR24-infected cells, suggesting that overexpression of DHCR24 slightly induced the activation of Akt. Consistent with this, treatment of Ad-DHCR24-infected cells with the Akt inhibitor IV following TM exposure significantly reversed the neuron-protective effect provided by the overexpression of DHCR24 (Fig. 6 E). Taken together, these data suggested that overexpression of DHCR24 improved the structural organization of caveolae, as well as the localization of IGF-1R, and thereby enhanced the activation of IGF-1-Akt survival signaling.

### Overexpression of DHCR24 Protects Neuronal Cells from Aβ-induced Apoptosis

**Figure 7:**
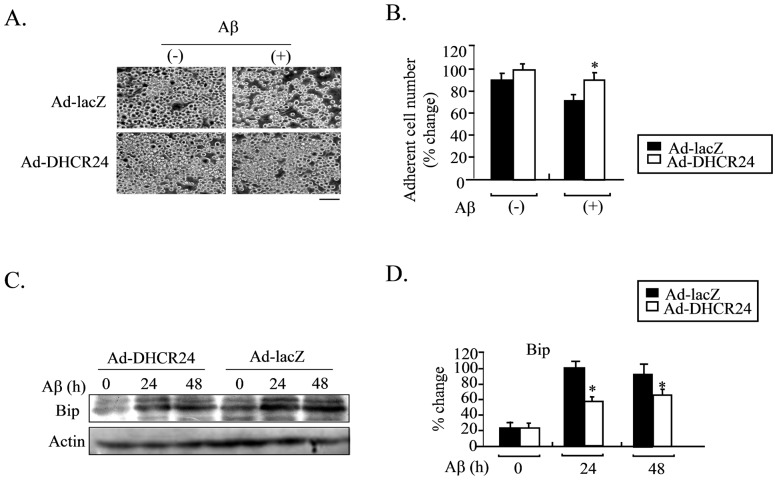
Ad-DHCR24-driven overexpression of DHCR24 protects neural cells from Aβ-induced apoptosis. Panel A and B, N2A cells were infected with the indicated adenoviral construct for 2 days, and then exposed to Aβ_1–42_ for 2 days_._ Images of the cells were obtained with a phase-contrast microscope at 48 hours after the Aβ treatment. Scale bar, 50 µm (Panel A). The number of adherent cells from panel A was counted at 0, 24 and 48 hours. Values are expressed as mean ± S.D. (n = 3) *p<0.05 vs. Ad-LacZ. Similar results were obtained from three separate experiments. Panel C and D, N2A cells were infected with the indicated adenoviruses for 2 days, and then exposed to Aβ_1–421_for 24 hours. Whole cell lysates (50 µg protein/lane) were subjected to Western blot analysis using anti-Bip and anti-actin antibodies. The proteins were visualized using the ECL method. Representative results are shown in Panel C. Bip levels were normalized against actin level, and are expressed as percentage of the levels of maximal levels in the cells infected with Ad-LacZ. n = 3, Mean ± S.D., *: p<0.05 vs. the levels in cells infected with Ad-LacZ.

Finally, we investigated the effects of DHCR24 overexpression on rescuing neuronal cells from Aβ-induced apoptosis. For both Ad-LacZ- and Ad-DHCR24-infected cells, the level of Bip was elevated at 24 and 48 hours following treatment with Aβ. This indicated that the ER stress occurred in response to Aβ. However, the induction of Bip in response to Aβ was more robust in Ad-LacZ-infected cells than in Ad-DHCR24-infected cells, both 24 and 48 hours after Aβ treatment (Fig. 7A and D). These results suggested that Aβ could induce ER stress in neuronal cells and overexpression of DHCR24 could suppress the induction of Bip induced by Aβ (Fig. 7 C and D).

## Discussion

The present study demonstrated that overexpression of DHCR24 in N2A cells (a neuronal cell line) could rescue the cells from ER stress-induced apoptosis (Fig. 1). The anti-apoptotic action of DHCR24 has been suggested by previous reports, including our own work [14], [16]. Greeve and *et al.* [14] reported decreased expression of DHCR24 in the brain area affected by Alzheimer’s disease. These investigators further showed that overexpression of DHCR24 protects neuronal cells from apoptosis induced by hydrogen peroxide and Aβ, strongly suggesting that DHCR24 may play a role in the scavenging of ROS produced by oxidative stress [14]. Recently, we demonstrated that DHCR24 protects MEFs from hydrogen peroxide-induced apoptosis through scavenging ROS in both *in vivo* and *in vitro* systems. We further showed that overexpression of DHCR24 can also protect MEFs from ER stress-induced apoptosis by scavenging the ROS generated by ER stress [17]. The result of the current study showed that DHCR24 also protected neuronal cells from ER stress-induced apoptosis.

DHCR24 suppressed ER stress-induced apoptosis through suppressing the activations of caspase-3 and caspase-12 (Fig. 2), suggesting that the ER stress-specific apoptotic signaling pathway was attenuated by DHCR24. Furthermore, induction of both Bip and CHOP, the two common ER stress-related molecular markers, was also reduced by the action of DHCR24 (Fig. 3), with the consequence of less severe stress levels in Ad-DHCR24-infected cells compared to control cells. This phenomenon was supported by the data obtained from western blot analysis using antibody against p-PERK, p-p38 or p-JNK (Fig. 4). This effect of DHCR24 on stress-related signaling pathway may be due in part to its ability to scavenge ROS, as well as its role in cholesterol biosynthesis. ROS generation in Ad-DHCR24-infected N2A cells was much weaker than in control N2A cells 3 hours and 24 hours after exposure to TM (Fig. 5), consistent with our previous study showing that DHCR24 can scavenge the ROS generated in MEFs under ER stress [17]. It is well accepted that oxidative stress and ER stress are the common pathological mechanisms in AD [32]. Aβ oligomers have recently been shown to accumulate in induced pluripotent stem cells (iPSC)-derived neurons and astrocytes from patients with a familial amyloid precursor protein (APP)-E693Δ mutation and sporadic AD, leading to ER and oxidative stress [33]. Here, we also demonstrated that overexpression of DHCR24 could protect N2A cells from hydrogen peroxide-induced apoptosis (data not shown). Together with the findings reported by other investigators, we believe that DHCR24 could protect neuronal cells from both oxidative stress and ER stress-induced apoptosis. This conclusion is further supported by the data presented in the present study (Fig. 7), which showed that Aβ oligomers could induce apoptosis and the expression of Bip in control N2A cells, whereas both events were suppressed in Ad-DHCR24-infected cells.

Recently, a new theory on AD has emerged, which considers AD as a third type of diabetes. This opinion is supported by a number of studies, especially since insulin-related cell survival signaling was shown to be downregulated in the affected areas of the brains of AD patients [34]–[36]. Additionally, many studies have reported abnormal insulin-signaling or insulin-like growth factor I (IGF-1) signaling pathways in AD-associated case studies or cellular experiments. Further support for this comes from clinical study revealing the effectiveness of long-trial intranasal insulin therapy for patients with amnestic mild cognitive impairment and patients with AD [37], [38]. DHCR24 protects MEFs from serum withdrawal-induced apoptosis through its function in cholesterol biosynthesis and maintenance of the structure of caveolae and insulin-Akt-Bad signaling [18]. We have also reported that IGF-1R localized in the caveolae in neuronal cells and cholesterol are necessary for the neuron-protective function of IGF-1 [31]. Therefore, we believe that DHCR24 could also protect neurons from apoptosis through its involvement in cholesterol biosynthesis and by facilitating the structural formation and function of caveolae. This belief was supported by the fact that the cholesterol level of N2As that overexpressed DHCR24 was significantly elevated compared to the cholesterol level of the controls (Fig. 6 A). In addition, overexpression of DHCR24 also facilitated the structure and the colocalization of IGF-1Rwith caveolae (Fig. 6 B and C). Taken together, our results strongly supported the function of DHCR24 in maintaining the structure of caveolae and facilitating the IGF-1-mediated cell survival signaling in neuronal cells.

In conclusion, the present study demonstrated that DHCR24 protected neuronal cells from ER stress-induced apoptosis, possibly through scavenging ROS and improving the function of membrane cholesterol. The neuron-protective function of DHCR24 is generally accepted, although its role as an AD indicator has recently been challenged. Through data obtained from microarray and RT-PCR experiments, Sharpe LJ and *at el.* [39] have pointed out that the expression of DHCR24 is not decreased in the affected brain area of AD patients. This is conflicting with an earlier report by Greeve and *at el*. [14]. These authors also suggested that the relationship between AD and DHCRC24 might be linked to its cholesterol biosynthesis function. We believe that regardless of the expression level of DHCR24 in AD patients, its neuroprotective function is apparent, possibly through multiple mechanisms, including cholesterol biosynthesis. The expression of DHCR24 is thought to decrease with ages. In the APPSLxPS1 mutant mouse model of AD, the levels of desmosterol in the cerebellum of nine-months-old animals were found to increase compared to control. The level of desmosterol in the hippocampus of 21-months-old mutant can increase by as much as 600% compared to the level of desmosterol in the control. By this age, there would be abundant deposits of amyloid, accompanied by decreased expression of DHCR24 [40]. Interestingly, in that particular study, some of the AD patients who carry certain DHCR24 polymorphism (rs718265 GG) have lower levels of Aβ_42_, indicating the DHCR24 gene may be associated with AD risk [41]. We believe that the reduced neuroprotective function of DHCR24 would arise as a result of either decreased expression of DHCR24 or polymorphism in the DHCR24 gene, either of which can be an important factor in the pathogenesis of AD.
